# Comparison of techniques for the evaluation of taste sensitivity

**DOI:** 10.1038/s41598-026-59454-2

**Published:** 2026-06-30

**Authors:** Jerry Hadi Juratli, I. Kaeb, A. Haehner, T. Hummel

**Affiliations:** Smell and Taste Clinic, Department of Otorhinolaryngology, Faculty of Medicine Carl Gustav Carus, Technische Universität Dresden, Fetscherstraße 74, 01307 Dresden, Germany

**Keywords:** Health care, Medical research, Neuroscience

## Abstract

Taste is essential for proper nutritional intake and thus has a significant effect on quality of life, yet clinical assessments of gustatory function are often underutilized or poorly standardized. This study aimed to compare two contrasting methodologies for evaluating taste sensitivity: Bayesian adaptive taste thresholds versus taste strip identification scores. One hundred participants underwent two sessions of gustatory testing, with a series of up to 20 trials of spray-based dilutions of sweet, sour, salty, and bitter tastants (QUEST) and the 16-item tastant-impregnated paper strips test. The log-transformed QUEST scores showed similar test–retest reliability to the taste strips score, with the bitter tastant showing the greatest correlation. There was no significant correlation between QUEST thresholds and taste strips identification scores. The QUEST spray test was rated more favorably by the participants for comprehensibility and pleasantness but took 12.38 min (± 33 s) on average, compared to 4.19 min (± 25 s) for the taste strips test. Tastant misidentifications on the taste strips assessment revealed that sour was disproportionately perceived as bitter at low concentrations but as salt at higher concentrations. QUEST logarithmic taste thresholds and tastant misidentifications on the taste strips test may serve as independent clinical measures of hypogeusia progression alongside taste strips identification score.

**Supplementary Information:**

The online version contains supplementary material available at 10.1038/s41598-026-59454-2.

## Introduction

The sense of taste plays a crucial role in nutrient sensing, modulating consumption of carbohydrates, sodium, and potentially toxic or spoiled foods. Taste dysfunction can affect the ability to detect nutrients and thus lead to malnutrition. Despite this significant role in daily life, and, in turn, its significant effects on quality of life in cases of taste dysfunction^1,2^, assessments of gustatory function are not universally available or applied in clinical settings.

Sensory testing is often measured through three different phenomena: (1) identification of a stimulus, (2) discrimination between different stimuli, and (3) sensitivity to a threshold concentration of a stimulus. For the latter, the threshold can be defined as either the detection threshold—concentration at which the subject can correctly detect the presence of a tastant—or the recognition threshold—concentration at which the tastant is correctly identified^3^.

In olfaction, odor threshold has been shown to explain more of the variability in overall Sniffin’ Sticks test battery score compared to discrimination and identification^4^. However, there is no direct equivalent gustatory threshold consistently used in clinical practice; taste identification, such as with tastant-impregnated taste strips, is a commonly-employed alternative^5–7^. Sensitivity to basic taste compounds can provide insight on eating behavior and diet^8^, with taste perceptual changes observed in obesity and metabolic disturbances^9,10^. Validated measures of taste threshold are clinically relevant for disordered eating as well as selective taste impairment.

The default approach to threshold testing is through the method of constant stimuli, wherein a fixed, complete ascending or descending range of stimuli are presented to all subjects, regardless of prior responses, over the entire psychometric function. In contrast, adaptive methods present stimuli differently to subjects based on their previous responses. Bayesian adaptive methods utilize the prior responses of a participant to approximate the predicted parameter value and deliver the next stimulus. QUEST is one such example, designed by Watson and Pelli to calculate stimulus strength based on a Bayesian adaptive measure of threshold^11^. When developing a taste threshold method, consideration should be given to the long inter-stimulus intervals required for gustation, given taste’s primarily phasic mechanism^12^. Starting testing at the expected threshold value will reduce the number of trials and overall length of testing, as well as reducing false positive/negative results^13^. A prior study implementing QUEST in odor threshold analysis found it comparable to the Sniffin’ Sticks odor threshold, itself an adaptive stepwise procedure^14^. In this context, utilizing the adaptive mechanism of QUEST may be a fitting method for a taste threshold assessment.

The present study aimed to evaluate the test–retest reliability of a spray-based QUEST method and the taste strips, and to compare threshold estimates between the two measures as well as trigeminal interactions via capsaicin. Furthermore, given its widespread use, more information was extracted from the 16-item taste strips assessment, including tastant misidentifications and tastant-specific thresholds. There are no existing analyses to our knowledge comparing QUEST taste threshold with taste identification in the assessment of taste sensitivity. Understanding the relationship between clinical tools for taste sensitivity can better aid phenotyping of gustatory dysfunction.

## Methods

### Participants

One hundred subjects (86 female, 14 male) from ages 33 to 57 were recruited into the study performed at the Department of Otorhinolaryngology of the TU Dresden for two identical visits typically 1 to 14 days apart. Exclusion criteria were pregnancy and test subjects who could not rule out pregnancy with certainty, neurological deficits, severe medical disorders, and known smell or taste disorders. Inclusion criteria for volunteers were subjectively normal smell and taste function, and age of 18 years and older. This study was conducted in accordance with the ethical principles of the Declaration of Helsinki for medical research involving human subjects and was approved by the Ethics Committee at the University Clinic of TU Dresden, Germany (BO-EK-556122022). Written informed consent was obtained from all participants prior to enrollment.

### Experimental sessions

Participants were instructed to abstain from eating, drinking, smoking, or brushing their teeth for 1 h before the visit. Subjects completed the WHO-5 depression screening (0 = low risk, 5 = high risk), as well as self-report of their sense of smell (0–5), taste (0–5), sensitivity against spiciness (0–4), and consumption of spicy food (0 = never, 4 = everyday).

The taste strips test consisted of 16 filter paper strips impregnated with one of four stimuli at four increasing concentrations (sucrose: 0.05, 0.1, 0.2, and 0.4 g/mL; citric acid: 0.05, 0.09, 0.165, and 0.3 g/mL; sodium chloride: 0.016, 0.04, 0.1, and 0.25 g/mL; quinine hydrochloride: 0.0004, 0.0009, 0.0024, and 0.006 g/mL). Concentrations are intended to be suprathreshold for normogeusic subjects. Strips were presented to participants in ascending order of concentrations within each tastant (lowest concentration for each stimulus first). Strips were placed on the anterior 2/3^rds^ tongue, and participants were asked to identify the taste quality perceived as sweet, sour, salty, or bitter. Possible scores for the whole test ranged from 0 to 16, with a range of 0 to 4 for each tastant.

### QUEST protocol

Tastants were formulated from sucrose (Sigma-Aldrich, CAS number: 57-50-1), citric acid (Sigma-Aldrich, CAS number: 77-92-9), sodium chloride (Sigma-Aldrich, CAS number: 7647-14-5), and quinine hydrochloride (Sigma-Aldrich, CAS number: 6119-47-7) dissolved in deionized water and stored at 4 °C. Solutions were brought to room temperature before testing. This formulation does not generate hazardous waste.

The QUEST threshold testing utilized logarithmically spaced concentration series for each tastant, spanning approximately four orders of magnitude. Concentration ranges in each dilution series were sucrose (0.025–200 mg/mL, 14 levels), citric acid (0.0029–9.0 mg/mL, 14 levels), sodium chloride (0.020–20.0 mg/mL, 12 levels), and quinine hydrochloride (0.00015–1.23 mg/mL, 18 levels). The QUEST psychometric method is initially described by Watson and Pelli^11^. Stimulus presentation in this study was performed as described by Höchenberger and Ohla^15,16^ in PsychoPy 1.85.4 in Python^17^.

Subjects were blindfolded and then informed about the taste to be identified in this block. The first stimulus for all subjects was a pre-established suprathreshold concentration. The solution was sprayed onto the anterior two-thirds of their outstretched tongue in a uniform pattern across trials and subjects, and subjects had to indicate whether they perceived the tastant or not before withdrawing their tongue. The QUEST procedure proposed the dilution level to present in subsequent trials based on all previous responses. There were 30 s between trials, and each test was repeated for up to 20 trials, after which the algorithm generates a threshold estimate for the tastant. This is repeated for each tastant, with three minutes between each analyte.

Following both the taste strips and QUEST tests, using Likert-type scales participants rated the pleasantness of the test (unpleasant = -5, pleasant =  + 5), their ability to concentrate on the test (1 = very easy, 4 = very hard), comprehensibility of the test (0 = very easy, 10 = very hard), handling of the test (0 = very easy, 10 = very hard), and motivation to complete the assessment (0 = very low, 10 = very high).

### Statistical analysis

#### Threshold test–retest reliability

Reliability of threshold estimates were assessed using multiple statistical and visual methods. Intraclass correlation coefficient (ICC) was the primary test for comparison of the consistency of different conditions. Wilcoxon signed-rank test and effect size were also used as a measure of comparison of reliability. Agreement of initial test and retest values were depicted using segment line plots generated in *ggplot* and Bland–Altman plots generated using the package *BlandAltmanLeh* in R. Paired t-tests were used to demonstrate agreement in intensity ratings to capsaicin capsules of four different concentrations.

#### Comparison of paper strips and spray threshold tests

Overall paper strips score were compared to log-adjusted QUEST threshold scores using general linear models (GLMs) and Pearson correlations. Wilcoxon signed-rank test and effect sizes were then utilized for comparison of threshold values between paper strips and QUEST spray for each tastant. Subjective evaluations—comprehensibility, sensation, motivation, handling, and ability to concentrate—on the given methods were compared using paired t-tests.

#### Tastant misidentification patterns

A chi-square (χ^2^) goodness of fit analysis was conducted on items within the 16-item taste strips test that had 15 or more incorrect responses from subjects, based on the assumption of an equal distribution of 1/3 for each incorrect answer if it were a purely random, uninformed guess.

## Results

### QUEST threshold vs taste strips identification score

**Figure 1:**
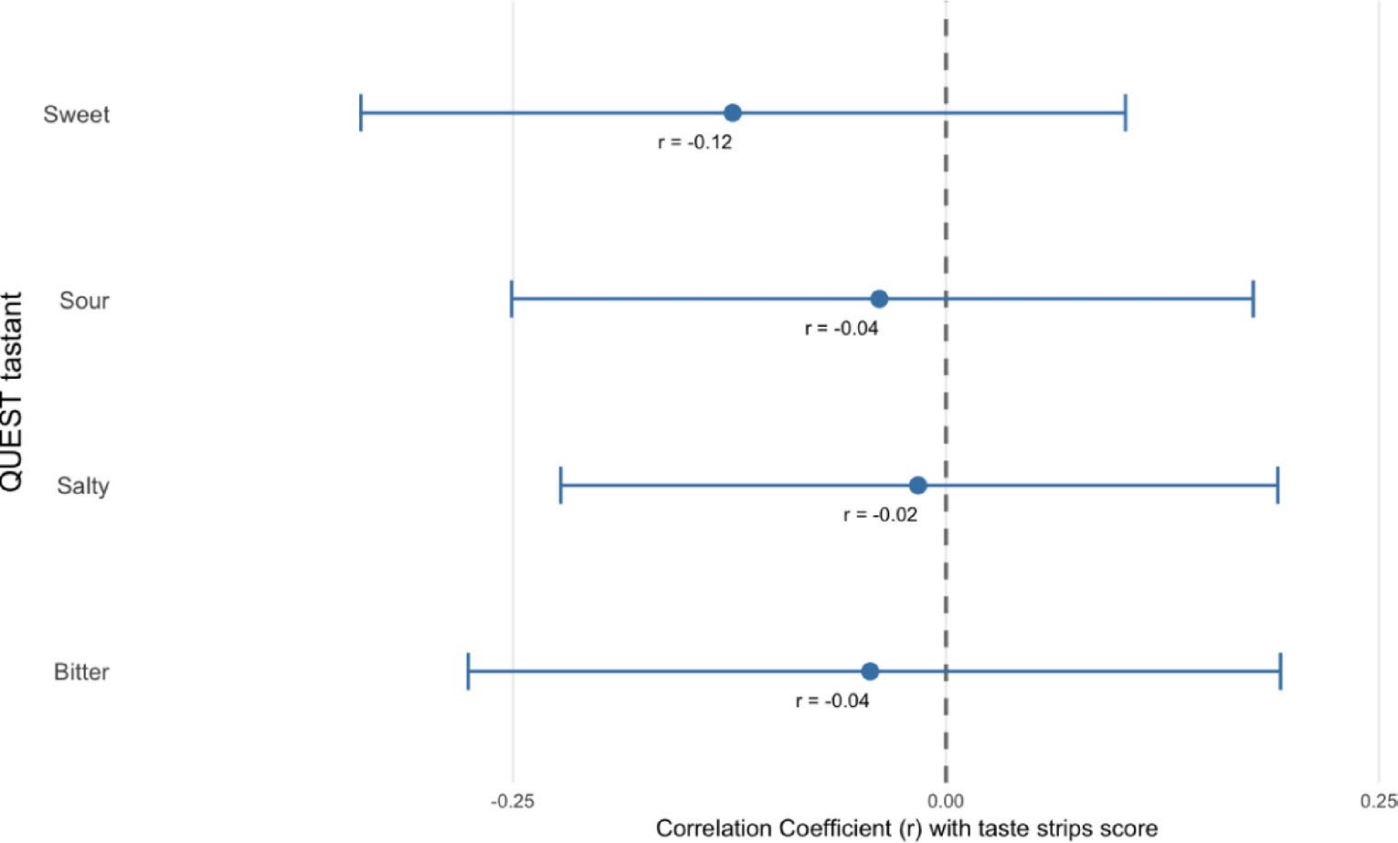
Forest plot of the correlation coefficients *r* between the taste strips identification score and the log-adjusted QUEST tastant thresholds. None of the correlations were statistically significant.

In a GLM of the paper strip test score as a function of the log-transformed scores on the four QUEST tests, citric acid (β = − 1.225, *p* = 0.0211) was a significant predictor of the paper strip score, whereas sucrose (β = − 0.349, *p* = 0.341), sodium chloride (β = − 0.800, *p* = 0.198), and quinine hydrochloride (β = 0.2515, *p* = 0.575) were not. The overall effect of the model was not significant (F(4,65) = 1.861, *p* = 0.128; R^2^ = 0.103). Tested independently, the taste strips identification score was not significantly correlated to any of the QUEST threshold scores (Fig. 1).

The QUEST test demonstrated favorable comprehensibility and pleasantness compared to the taste strips assessment, although both were ranked positively on average in all metrics (Table 1). The paper strips 16-item assessment took 4.19 (± 25 s) minutes on average, compared to 12.38 (± 33 s) minutes for the QUEST spray test (t = − 53.63, *p* < 0.0001, Cohen’s d = 5.36).

**Table 1:** Subject evaluation of the QUEST spray and taste strips assessments.

Variable	Test	Mean score (± SE)	Bonferroni-adjusted p value
Comprehensibility (0 [very easy]—10 [very hard])	Paper	1.16 (± 0.18)	0.0143
	Spray	0.67 (± 0.09)	
Concentration on the test (0 [very easy]—4 [very hard])	Paper	2.17 (± 0.08)	1
	Spray	2.03 (± 0.08)	
Handling (0 [very easy]—10 [very hard])	Paper	0.66 (± 0.09)	1
	Spray	0.72 (± 0.10)	
Motivation to complete test (0 [very low]—10 [very high])	Paper	8.90 (± 0.15)	1
	Spray	8.85 (± 0.15)	
Sensation of stimuli (-5 [very unpleasant]—5 [very pleasant])	Paper	1.05 (± 0.23)	0.0001
	Spray	2.21 (± 0.21)	

### QUEST taste spray test–retest reliability

ICC were calculated for the log-transformed values for each QUEST spray test to assess test–retest reliability. ICC(A,1) was 0.515 for sucrose [95% CI (0.314, 0.668)], 0.486 for citric acid [95% CI (0.241, 0.660)], 0.488 for sodium chloride [95% CI (0.314, 0.631)], and 0.567 for quinine hydrochloride [95% CI (0.363, 0.714)], indicating that the results for the four tastants were moderately reliable (Table 2). This degree of confidence mirrors that of the established paper strips test, which had an ICC(A,1) = 0.662 [95% CI (0.523, 0.764)].

**Table 2:** Mean thresholds for taste sprays calculated by QUEST, with test–retest reliability following log transformation of threshold values calculated by ICC. Visits where the threshold value fell below the detectable range were excluded.

Test	Tastant	n1, n2	Test mean threshold (± SE)	Retest mean threshold (± SE)	ICC(A,1) following log transformation
Spray	Citric acid (mg/mL)	89, 90	10.93 (± 1.66)	4.42 (± 0.67)	0.486
	Quinine hydrochloride (mg/mL)	79, 78	0.809 (± 0.374)	0.264 (± 0.082)	0.567
	Sodium chloride (mg/mL)	93, 91	12.35 (± 1.66)	9.46 (± 1.25)	0.488
	Sucrose (g/mL)	87, 78	0.681 (± 0.116)	0.275 (± 0.048)	0.515

**Figure 2:**
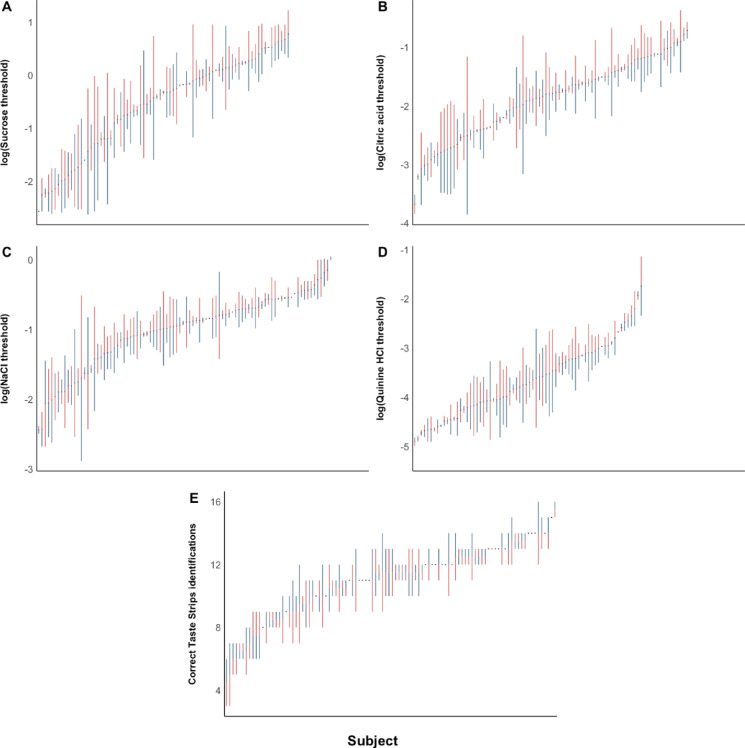
**(A–E)** Segment interval plots between two test sessions for all participants, excluding those where the threshold value fell below the detectable range. Each vertical line indicates a participant, with the red segment indicating the first score and the blue segment indicating the retest score, emanating from the mean, represented as a black dot. Subjects are ordered by mean average score across the two visits.

**Figure 3:**
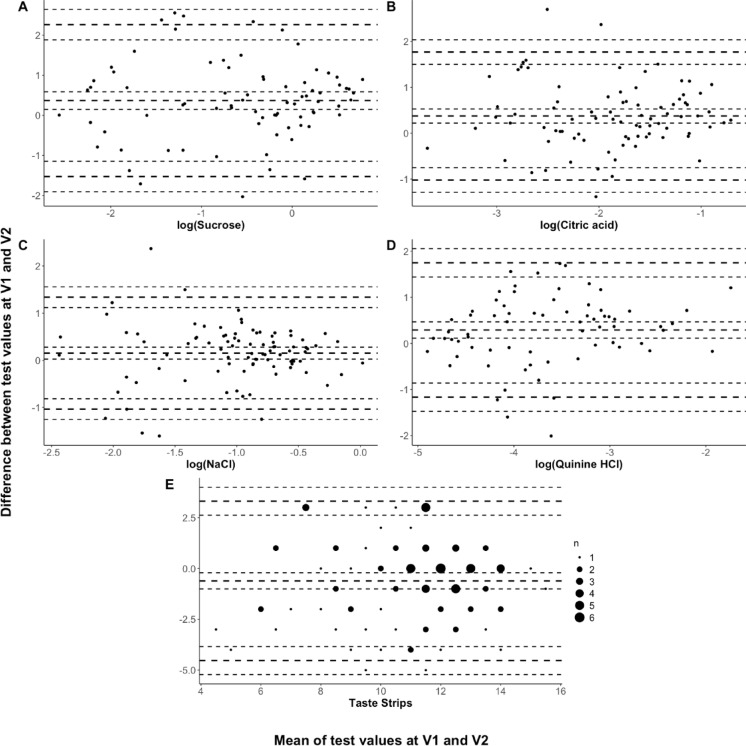
**(A–E)** Bland–Altman plots of the threshold test scores of each subject at the first and second visit (V1 and V2, respectively) for the QUEST spray test (A-D) and the taste strips (E). The centermost line represents the bias (mean difference) between test and retest values. Upper and lower limits of agreement are located 1.96 standard deviations from the mean line. The limits of agreement also have a 95% confidence interval, represented by shorter dashed lines. There are few outliers outside of the upper and lower limits across all threshold tests.

There was weak to no correspondence in the calculated test–retest threshold values (mg or g/mL) from the QUEST method, with the ICC ranging from 0.0144 (bitter) to 0.296 (sweet). Participants consistently had statistically significant lower scores for the log-transformed QUEST thresholds and paper strip tests during the second session, but with small effect sizes (r < 0.2 for all; Figs. 2, 3).

### Tastant misidentification patterns

Misidentification of the tastant on the paper strips can suggest that the subject either (a) could not perceive any gustatory stimulus, or (b) attributes the properties of one tastant to another. If the former, then the three possible wrong choices would be selected in approximately equal proportions.

Of the 16 paper strips utilized, 11 were misidentified more than 15 times, resulting in an expected distribution of at least 5 for each incorrect answer. The excluded tastants (erroneously identified by < 15 subjects) include the two highest concentrations for sweet (0.2 g and 0.4 g) and bitter (0.0024 g and 0.006 g) and the highest concentration for salty (0.25 g). At least half of the subjects misidentified all but the highest sour concentration. As expected, there was greater identification accuracy at higher concentrations for all tastants (Table 3).

**Table 3:** Tastant misidentification distributions for taste strips misidentified in n > 15 trials.

Tastant	Mass (g)	n	“Sweet” (%)	“Sour” (%)	“Salty” (%)	“Bitter” (%)	χ^2^	Non-adjusted* p*-value
Bitter	0.0004	53	13.2	69.8	17.0		31.9	< 0.0001*
	0.0009	32	9.4	65.6	25.0		17.3	0.00018*
Salty	0.016	36	5.6	72.2		22.2	26	< 0.0001*
	0.04	29	10.3	69.0		20.7	17.0	0.0002*
	0.1	30	10.0	56.7		33.3	9.8	0.00745
Sour	0.05	88	15.9		19.3	64.8	39.3	< 0.0001*
	0.09	89	16.9		36.0	47.2	12.6	0.00187*
	0.165	50	6.0		58.0	36.0	20.4	< 0.0001*
	0.3	32	0.0		65.6	34.4	20.7	< 0.0001*
Sweet	0.05	34		26.5	35.3	38.2	0.76	0.68
	0.1	19		26.3	42.1	31.6	0.74	0.69

**Figure 4:**
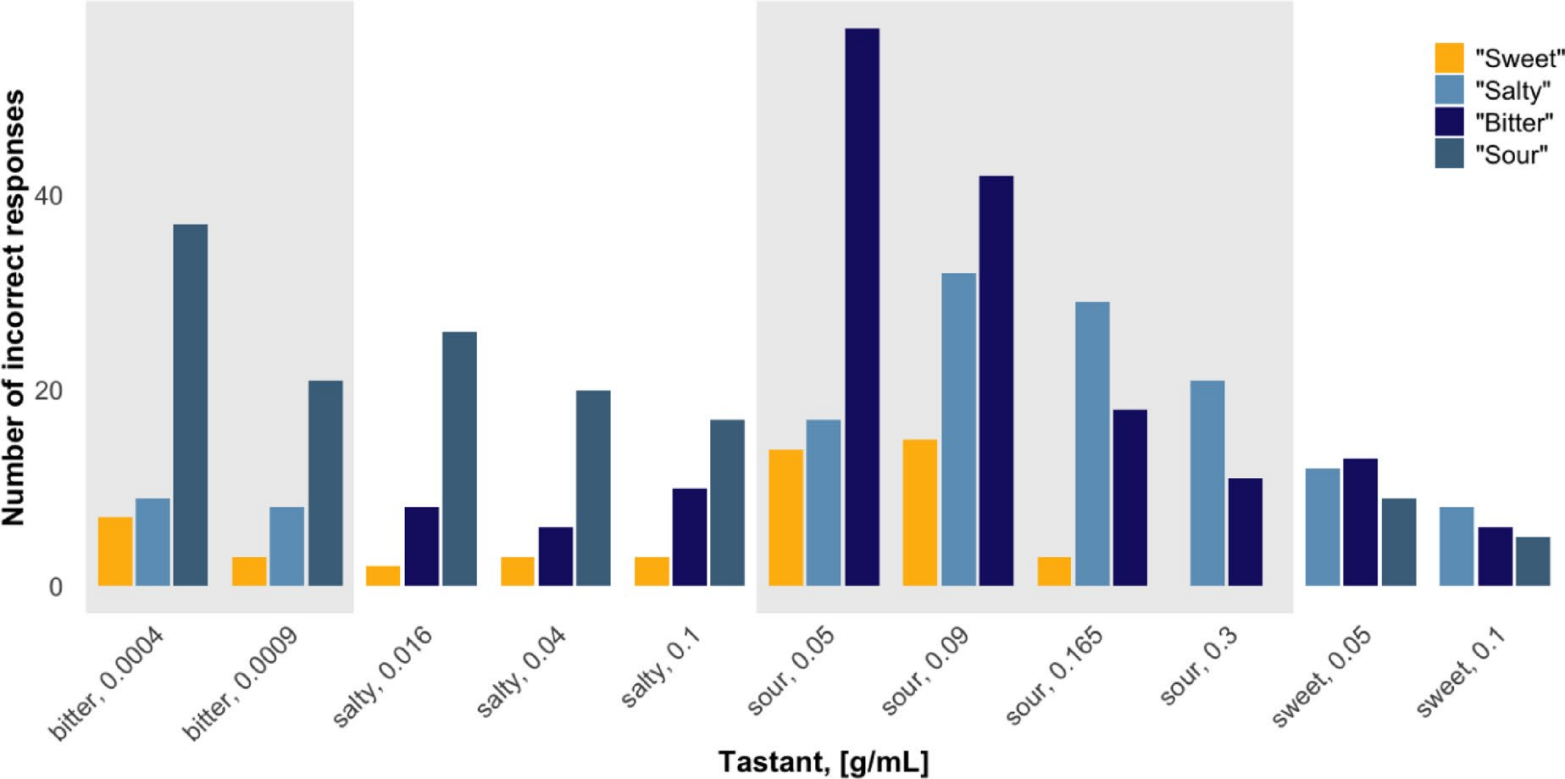
Frequency plot of tastant misidentification patterns for 11 items from the 16-item taste strips task.

Chi-square goodness of fit testing was conducted assuming an expected distribution of 1/3 for each incorrect option. Of the 11 tastant concentrations, eight were significantly different relative to the Bonferroni-adjusted alpha value α_adj_ = 0.00455. Bitter and salty were both most misidentified as sour and least often as sweet. At the lower two concentrations, sour was more commonly misattributed as bitter, but at higher concentrations was largely attributed as salty. The highest two concentrations of sour were increasingly less likely to be identified as sweet, in comparison to the lower concentrations (Fig. 4). Misidentifications for sweet closely followed the expected distribution, evenly split across the three remaining tastants of sour, salty, and bitter.

## Discussion

### Threshold repeatability and validity

The log-transformed values from QUEST taste spray method showed test–retest reliability on par with the validated taste strips. Prior studies have also shown similar reliability of the QUEST method with other protocols, including quick Yes–No (qYN)^16^ and single-interval adjustment-matrix (SIAM)^15^. Interestingly, both studies and the present analysis found that Quinine-HCl demonstrated the highest test–retest correlation, and citric acid—albeit marginally—the lowest among the QUEST tastants^15,16^. This may indicate that bitter compounds, in a spray-based assessment, are a more reliable measure of changes in gustatory function and have greater clinical utility in diagnosing gustatory impairments. Taste sensitivity to bitter was also the lowest by orders of magnitude compared to the other tastants, a finding consistent with established knowledge on taste percepts in humans^18^. Thus, when assessing taste threshold, bitter may serve as a more reliable measure due to our increased sensitivity.

Importantly, QUEST threshold values showed good test–retest reliability only once log-transformed, which can be used to track hypogeusia progression or compare to normative values, rather than predicting an exact threshold for each tastant. There is precedent for the clinical use of log-adjusted values of sensory thresholds (e.g., logMAR for visual acuity, dB for sound intensity)^19^. This is consistent with Fechner’s law, which posits that perception increases logarithmically with stimulus concentration^20^. Accordingly, log-transformed QUEST thresholds better align with the underlying psychophysical scale of gustatory perception, thereby reducing variability and improving test–retest reliability.

A limitation of QUEST is that while the yes–no paradigm is appealingly straightforward, alternative forced choice (AFC) is widely known to be more reliable in equal conditions^21^. The use of yes–no in QUEST does not control the response criterion and can result in a higher proportion of “yes” responses. Despite the simplified paradigm, average threshold values improved at retest for all tastants, suggesting the presence of a learning effect. Future applications of the spray threshold test may mitigate this with pre-test training or increased trials.

Beyond the presence of a learning effect, the test–retest variability in taste sensitivity reflects an established challenge in gustatory testing. A major issue is reliable stimulus control: characteristics of both the stimulus and the experimental design must be accounted for, including pH, temperature, timing, and location of stimulus presentation. Crucially, the stimulus must achieve a steep rise and fall in percept with a sufficient washout period, which is challenging when paired with the high concentrations needed to elicit a response^22^. Thus, consistent measures of taste sensitivity rely on advances in gustatory stimulation.

### Taste sensitivity comparisons

Scores on the QUEST taste threshold were not correlative with the taste strips identification task. This could be expected given the tests measure different phenomena (threshold vs identification). Furthermore, the taste strips test utilizes tastant concentrations that are suprathreshold for most subjects^6^. The dissociation between threshold and identification performance also reflects distinct underlying mechanisms. Threshold detection primarily engages basic sensory processing and signal detection capabilities, whereas identification tasks additionally require cognitive components^23^. This distinction is analogous to differences observed in olfactory testing, where detection and identification tasks often show divergent patterns of impairment across clinical populations^24^.

Route of administration (impregnated paper strips vs diluted sprays) also contributes to the observed difference. Taste strips were designed to be selectively placed on regions of the tongue and could be lateralized to the left or right of the anterior third of the tongue, in addition to whole-mouth assessment^6^. Perceived intensity of sweet strips have also been found to significantly differ when applied across the tongue or to the tip of the tongue^25^. While administration of the taste strips and spray were controlled in this study, variations in tongue anatomy may favor a diffuse tastant distribution for clinical assessment.

### Taste misidentifications

Low concentrations of sour were disproportionately perceived as bitter; at higher concentrations, it was instead confused with salt. In contrast, both salt and bitter are perceived as sour at lower concentrations. Sweet is not significantly mistaken for one tastant over others.

These patterns are generally supported by observations from a study by Welge-Lüssen and colleagues, which reported misidentifications for the highest concentrations of each tastant, albeit without statistical validation^26^. They found that sweet was not consistently mistaken for a particular taste; sour and salty were commonly mistaken for each other, followed by bitter; and bitter was mostly confused for sour^26^. Using aqueous solutions, Pilková and colleagues also found that sucrose (20.0 g/l) was the most often correctly identified tastant, and in cases where sodium chloride (5.0 g/l) was incorrectly identified, it was commonly labeled as sour^27^. A whole-mouth taste test found that sour-bitter confusions were most common, with confusions for sweet stimuli the least common^28^. Future analyses may investigate if rarer taste confusions (e.g., involving sweet stimuli) correspond to severity of taste dysfunction.

These patterns can be explained partially by sensory encoding patterns. Sour and salty receptors are both transient receptor potential ion channels, as opposed to GPCRs for sweet, bitter, and umami^29,30^. This electrochemical pathway can explain overlap of sour and salty perception at low concentrations; this may be a similar mechanism to how electrogustometry corresponds with a salty sensation^31,32^. Furthermore, a study of parageusic patients found common reports of salty sensations, as well as mixed bitter-salty or sour–sweet phenomena^33^. While all subjects in this study had healthy gustatory function, the mechanisms of parageusia/phantogeusia present a possible explanation for misidentifications. Studies comparing cohorts or patient populations could utilize incorrect answers from the taste strips assessment to identify whether the group has a higher incidence of quantitative gustatory loss (random distribution of incorrect answers) or a qualitative gustatory dysfunction (significant false attribution of a tastant to an unrelated taste). Furthermore, the frequent misidentifications of citric acid may suggest that it is a good indicator of parageusia but less suited as a measure of taste sensitivity.

## Conclusions

The present study found that QUEST logarithmic taste thresholds demonstrate good test–retest reliability and can serve as a clinical measure of hypogeusia progression alongside taste strips identification score. Furthermore, tastant misidentifications in the taste strips test may be used to distinguish hypogeusia from complete ageusia at the group level.

## Supplementary Information

Below is the link to the electronic supplementary material.

Supplementary Material 1.

## Data Availability

The data that support the findings of this study are available from the corresponding author, JJ, upon reasonable request.
