# Supplementary material for: Comparison of techniques for the evaluation of taste sensitivity
Source: Sci Rep. 2026 Jun 30;16:19919. doi: 10.1038/s41598-026-59454-2 (PMC13319474; doi:10.1038/s41598-026-59454-2)
Supplement: Supplementary file 1 — Supplementary Material 1. [file 41598_2026_59454_MOESM1_ESM.docx]

**SUPPLEMENTARY MATERIAL**

**Table S1.** Wilcoxon signed-rank test of test-retest scores for the log-transformed spray thresholds and paper strips identification scores.

| **Test** | **Tastant** | ***V*** | **Bonferroni-adjusted *p*-value** | **Effect size *r*** |
| --- | --- | --- | --- | --- |
| log(Spray) | Citric acid | 3467.0 | 0.0001272 | 0.17 |
|  | Quinine hydrochloride | 3178.0 | 0.0034560 | 0.10 |
|  | Sodium chloride | 3190.0 | 0.0029440 | 0.15 |
|  | Sucrose | 3342.0 | 0.0000378 | 0.17 |
| Paper |  | 868.5 | 0.014700 | 0.12 |

**Table S2.** GLM displaying that sex of the participant did not have a significant effect on the scores for each taste test.

| **Test** | **Variable** | **n** | **Sex (F=0, M=1)** |  |
| --- | --- | --- | --- | --- |
| Spray | log(Citric acid) | 89 | β = 0.328  *p* = 0.167 |  |
|  | log(Quinine hydrochloride) | 79 | β = 0.408  *p* = 0.188 |  |
|  | log(Sodium chloride) | 93 | β = 0.188  *p* = 0.351 |  |
|  | log(Sucrose) | 87 | β = 0.528  *p* = 0.112 |  |
| Paper |  | 100 | β = -0.017  *p* = 0.165 |  |

**Threshold comparison between paper and spray tests**

While the taste strips are primarily leveraged as a measure of taste identification, the simultaneous use of four concentrations for each tastant allows for the calculation of a quasi-threshold for taste recognition. This corresponds to the lowest concentration guessed correctly, assuming the higher concentrations were also answered correctly. If three out of four were correct, of which the lowest concentration was included, then the threshold was judged as equal to that of the lowest concentration. If a concentration and all subsequent higher ones were answered correctly, then the lowest correctly answered concentration was assessed as the “threshold”. In cases with a gap between two correct answers where the highest concentration was correct, the threshold was estimated as the midpoint between the two concentrations.

The calculated paper strip thresholds demonstrated good test-retest reliability through the Wilcoxon test with Bonferroni correction. The values for sweet (V = 469, *p* = 0.268), salty (V = 481, *p* = 0.108), and sour (V = 335, *p* = 0.526) were consistent across the first and second visits. Bitter threshold was statistically different at retest (V = 677.5, *p* = 0.0170), but with a very small effect size (r = 0.047).

Agreement between the paper strips and spray thresholds was assessed with a paired Wilcoxon test only in subjects with a paper strips threshold. Furthermore, there were no significant correlations between the two tests for bitter (r = 0.041), sour (r = 0.21), salty (r = -0.13), or sweet (r = 0.045).

Paper strips seemingly produced reliable taste threshold estimates with no significant differences in the test-retest reliability across tastants except for bitter, in contrast with QUEST which found significant changes in threshold at retest. However, taste strip thresholds effectively took on discrete values, with only four concentrations tested per tastant. Furthermore, subjects whose threshold could not be determined by the four paper strips (ranging between 3 and 31 subjects for sweet and sour, respectively) were excluded from the analysis. Therefore, the data is inherently more consistent, and the veracity of the threshold estimates is not directly applicable. Calculation of recognition thresholds from the Taste Strips method requires further analysis with an extended set of concentrations to validate its accuracy.

**Table S3.** Comparison of calculated thresholds from the paper strips and QUEST spray methods for participants with valid values for both.

| **Tastant** | **Test** | **n** | **Mean threshold ± SE (g/mL)** | **V** | **Adjusted p-value** | **Effect size *r*** |
| --- | --- | --- | --- | --- | --- | --- |
| bitter | paper | 73 | 0.00147 ± 0.000210 | 2334 | <0.001 | 0.536 |
|  | spray | 73 | 0.000464 ± 0.000107 |  |  |  |
| salty | paper | 81 | 0.0559 ± 0.00834 | 2873 | <0.001 | 0.530 |
|  | spray | 81 | 0.0122 ± 0.00129 |  |  |  |
| sour | paper | 61 | 0.197 ± 0.0104 | 1891 | <0.001 | 0.864 |
|  | spray | 61 | 0.0111 ± 0.00219 |  |  |  |
| sweet | paper | 84 | 0.100 ± 0.00989 | 710 | <0.001 | 0.213 |
|  | spray | 84 | 0.701 ± 0.120 |  |  |  |

**Table S4.** Test-retest reliability of paper strip taste thresholds

| **Test** | **variable** | **n** | **Test mean value (± SE) g/mL** | **Retest mean value (± SE) g/mL** | ***V*** | **Bonferroni-adjusted *p*-value** | **Effect size *r*** |
| --- | --- | --- | --- | --- | --- | --- | --- |
| Paper | Bitter | 93 | 0.00151 (± 0.000186) | 0.00119 (± 0.000157) | 677.5 | 0.01696 | 0.047040418 |
|  | Salty | 86 | 0.0569 (± 0.00820) | 0.0465 (± 0.00619) | 481.0 | 0.43200 | N/A |
|  | Sour | 69 | 0.195 (± 0.00982) | 0.187 (± 0.00855) | 335.0 | 1.00000 | N/A |
|  | Sweet | 97 | 0.0954 (± 0.00878) | 0.0921 (± 0.00953) | 469.0 | 1.00000 | N/A |
